# Relationship Between Cerebrovascular Diseases and Vasculitis: A Cross-Sectional Nationwide Inpatient Study

**DOI:** 10.7759/cureus.27435

**Published:** 2022-07-29

**Authors:** Anusheel ., Johanna S Canenguez Benitez, Sanobar Jaka, Nikhita S Roshan, Sravani Kommuru, Samreen Ahmed, Gagan Kaur, Ninad Desai

**Affiliations:** 1 Medicine, Shanti Gopal Hospital, Ghaziabad, IND; 2 Internal Medicine, Larkin Community Hospital, South Miami, USA; 3 Epidemiology and Public Health, Psychiatry, New York University (NYU) School of Global Public Health, New York, USA; 4 Neurology, Father Muller Medical College, Mangalore, IND; 5 Internal Medicine, Dr. Pinnamaneni Siddhartha Institute of Medical Sciences and Research Foundation, Vijayawada, IND; 6 Neurology, University of Illinois, Chicago, USA; 7 Medicine, Sri Guru Ram Das Institute of Medical Sciences and Research, Amritsar, IND; 8 Neurology, St. Vincent's Medical Center, Bridgeport, USA

**Keywords:** hypertension, risk factors, hospitalization outcomes, cerebro-vascular accident (stroke), cerebral vasculitis

## Abstract

Objectives

To evaluate the risk factors and hospitalization outcomes for cerebrovascular diseases (CVD) in patients with vasculitis.

Methods

We conducted a cross-sectional study using the Nationwide Inpatient Sample (NIS), 2019. We included 26,855 adults (aged 18 to 65 years, average age 48.57 ± 12.79 years) with a co-diagnosis of vasculitis, and the sample was divided by the primary diagnosis of CVD (N = 670, 2.5%). A demographic-adjusted logistic regression model was used to evaluate the odds ratio (OR) of association with CVD in patients with vasculitis by comparing it to the non-CVD cohort.

Results

The majority of the vasculitis patients with CVD were elders (51 to 65 years, 46%), females (62%), and whites (52%). There was a significant difference in the geographic distribution of CVD with vasculitis with the highest prevalence in the South Atlantic (23%) and Middle Atlantic (16%), and the lowest in the Mountain (4%) and New England (2%). Vasculitis patients with comorbid lymphoma (OR 2.46, P<0.001), peripheral vascular diseases (PVD (OR 1.54, P<0.001)), and complicated hypertension (OR 1.31, P<0.001) were associated with increasing the likelihood for CVD-related hospitalization. The mean length of stay was 13 days and the mean cost was $169,440 per CVD-related hospitalization in vasculitis patients. Cerebrovascular diseases in patients with vasculitis resulted in a major loss of body functioning (80%) leading to adverse disposition including transfer to a skilled nursing facility/intermediate care facility (22%) and requiring home health care (13%).

Conclusion

The prevalence of CVD-related hospitalization in vasculitis patients was 2.5% and females were observed to be at higher risk. Comorbid lymphoma, PVD, and hypertension further increase the risk for CVD with vasculitis. They have a higher loss of functioning that affects patient quality of life and require increased care after hospital discharge.

## Introduction

A cerebrovascular accident occurs every 40 seconds in the United States (US) [1]. About 7.8 million of the adult American population have experienced a cerebrovascular accident [2]. The risk for cerebrovascular disease (CVD) varies with race and ethnicity. It is nearly twice as high for the Black community compared to Caucasians, and Black people have the highest rate of death due to CVD [3]. The mortality rate due to CVD has also risen among the Hispanic population since 2013 [3]. The risk for CVD increases with age; 10% to 15% of all cerebrovascular accidents occur among 18 to 50 years old adults [4,5]. The incidence and prevalence of cerebrovascular accidents are dramatically increasing regardless of socioeconomic strata [6]. Around 80% of cerebrovascular accidents in the young population are known to be ischemic and 20% are hemorrhagic [6]. However, multiple studies have studied the risk factors causing a dramatic increase in CVD among the young population and have found overlapping traditional risk factor associations [5,6]. These include hypertension, diabetes mellitus, dyslipidemia, and smoking among other well-known risk factors [6]. In the US, CVD affects males more often, but the mortality rate is higher in females [5]. The CVD-related costs of care including medicines and loss of days from work were calculated to be nearly $53 billion between 2017 and 2018 in the US. [4]. Cerebrovascular diseases are more prevalent within the south-eastern states of the US, and in areas with extreme climates [5]. 

Central nervous system (CNS) vasculitis can be primary or secondary involving other systems [7]. Primary angiitis of the CNS (PACNS) commonly presents as stroke, encephalopathy, and recurrent headaches [7]. Giant cell arteritis (GCA) affects large vessels whereas medium-sized arteries are involved in polyarteritis nodosa (PAN) [7]. Although in general, vasculitis is rare, the prevalence of medium and small vasculitis has increased in the last decade, likely due to advancements in the treatment options such as prednisone and cyclophosphamide [8,9]. Cerebral angiitis is uncommon and not easily identified but can be fatal. Cerebrovascular disease in the young population can be a sequela of cerebral vasculitis [10]. Pathogenesis behind thrombosis has been extensively studied and there is evidence of chronic inflammation causing increased vascular thrombosis in autoimmune diseases including vasculitis in turn triggering the coagulation cascade [11,12]. This could explain the higher risk for CVD in patients with vasculitis [13]. Of all types of vasculitis, patients with primary central nervous system angiitis have the highest risk (40%) of developing CVD [14]. About 6% of patients with systemic necrotizing vasculitis and 3% to 7% of patients with GCA developed CVD while over 10% of patients with Takayasu arteritis (TA) experienced ischemic stroke [11,15,16]. There is limited data and inadequate literature on risk factors that may predispose CVD in vasculitis patients.

Thus, we aimed to study demographic factors and comorbidities that increase hospitalization due to CVD in vasculitis patients. Additionally, we analyzed the length of stay (LOS) and cost of care due to CVD hospitalization and studied the severity of illness, in-hospital mortality, and disposition in vasculitis inpatients. Thereafter, we established the prevalence of CVD with vasculitis across US geographical regions.

## Materials and methods

Study sample

We conducted a retrospective cross-sectional study using the Nationwide Inpatient Sample (NIS) from Jan 1, 2019, to Dec 31, 2019 [17]. The NIS is the inpatient database from the non-federal community hospitals across 48 states, and the District of Columbia in the US. As the NIS is a publicly available secured de-identified database, it does not require approval from an institutional review board according to the Agency for Healthcare Research and Quality (AHRQ) and the Department of Health and Human Services [17]. We included 26,855 adult inpatients (aged 18 to 65 years) hospitalized on a non-elective admission basis with a co-diagnosis of vasculitis. The study sample was divided by the presence of a primary diagnosis of CVD that includes middle cerebral artery syndrome, anterior cerebral artery syndrome, posterior cerebral artery syndrome, brain stem stroke syndrome, cerebellar stroke syndrome, pure motor lacunar syndrome, and/or pure sensory lacunar syndrome. The Clinical Classifications Software Refined (CCSR) for International Classification of Diseases (ICD-10) diagnoses aggregates more than 70,000 ICD-10 diagnosis codes into over 530 clinical categories in the NIS. We used CCSR code CIR037 from the circulatory system to identify vasculitis [18]. We used CCSR code CIR020 for cerebral infarction, CIR021 for acute hemorrhagic cerebrovascular disease, CIR022 for sequela of hemorrhagic cerebrovascular disease, CIR023 for occlusion or stenosis of precerebral or cerebral arteries with infarction, CIR024 for other and ill-defined cerebrovascular disease, and CIR025 for sequela of cerebral infarction and other cerebrovascular diseases to identify CVD [18].

Variables

The variable of interest included demographic characteristics (age, sex, and race), and comorbidities which are the co-diagnoses in the patient records and we included arthropathies, lymphoma, metastatic cancer, diabetes with complications, hypertension (complicated), obesity, drug abuse, and peripheral vascular diseases (PVD). We included geographical areas as represented in the NIS by the nine US Census Bureau i.e., New England (Maine, New Hampshire, Vermont, Massachusetts, Rhode Island, Connecticut), Middle Atlantic (New York, Pennsylvania, New Jersey), East North Central (Wisconsin, Michigan, Illinois, Indiana, Ohio), West North Central (Missouri, North Dakota, South Dakota, Nebraska, Kansas, Minnesota, Iowa), South Atlantic (Delaware, Maryland, District of Columbia, Virginia, West Virginia, North Carolina, South Carolina, Georgia, Florida), East South Central (Kentucky, Tennessee, Mississippi, Alabama), West South Central (Oklahoma, Texas, Arkansas, Louisiana), Mountain (Idaho, Montana, Wyoming, Nevada, Utah, Colorado, Arizona, New Mexico), and the Pacific (Alaska, Washington, Oregon, California, Hawaii) [17].

The hospitalization outcomes of interest included: severity of illness, disposition, and in-hospital mortality (all-cause). The severity of illness was evaluated using the all-patient refined diagnosis-related groups (APR-DRGs) which were developed by 3M Health Information Systems (Murray, UT, USA). Disposition of the patient at discharge in the NIS was classified as routine, transfer to short-term hospitals, transfers to other facilities like skilled nursing facilities (SNF) and intermediate care facilities (ICF), transfer to home health care, and discharge against medical advice [17].

Statistical analysis

We used Pearson's chi-square test and independent-sample t-test for categorical data and continuous data, respectively. Descriptive statistics were used to evaluate the differences in demographics and hospitalization outcomes between CVD and non-CVD cohorts. A binomial logistic regression model was used to evaluate the odds ratio (OR) of the association for CVD in inpatients with vasculitis compared to the non-CVD cohort (i.e., reference category). A p-value <0.01 was used to determine the statistical significance and all statistical analyses were conducted using the Statistical Package for Social Sciences (SPSS) version 27 (IBM Corp., Armonk, NY, USA).

## Results

We included 26,855 adult inpatients with vasculitis that majorly constituted elders (51 to 65 years, 53.4%), males (57%), and whites (58.9%). The prevalence of CVD in vasculitis inpatients was 2.57% (N = 690). The majority of vasculitis inpatients with CVD were elders (51 to 65 years), females (61.9%), and whites (51.9%). And, when compared to the non-CVD cohort, a significantly higher proportion of inpatients in the CVD cohort were middle-aged adults (33.6% vs. 27.5%), Black people (27.5% vs. 19.3%), and Hispanics (16% vs 14.3%). As far as the co-morbidities are concerned, the most prevalent were PVD (55.2%) and arthropathies (55.2%), followed by hypertension (44%), obesity (17.9%), and diabetes (16.4%) in vasculitis inpatients with CVD as shown in Table 1.

**Table 1:** Differences in demographic characteristics in vasculitis inpatients CVD: Cerebrovascular disease

Variable	CVD (no) in %	CVD (yes) in %	Total in %	P-value
Age at admission				
18-35 years	18.9	20.1	19.0	<0.001
36-50 years	27.5	33.6	27.6	
51-65 years	53.6	46.3	53.4	
Sex				
Male	43.1	38.1	43.0	0.009
Female	56.29	61.9	57.0	
Race/ethnicity				
White	59.0	51.9	58.9	<0.001
Black	19.3	27.5	19.5	
Hispanic	14.3	16.0	14.4	
Other	7.3	4.6	7.3	
Comorbidities				
Arthropathies	67.4	55.2	67.1	<0.001
Lymphoma	1.0	2.2	1.0	0.002
Metastatic cancer	1.9	1.5	1.9	0.399
Diabetes with complications	22.2	16.4	22.0	<0.001
Hypertension, complicated	36.2	44.0	36.4	<0.001
Obesity	22.0	17.9	21.9	0.012
Drug abuse	8.5	9.0	8.5	0.662
Peripheral vascular diseases	41.6	55.2	41.9	<0.001

According to the national data analyzed in this study, the population affected with CVD-related hospitalization in patients with vasculitis is located mainly in the South Atlantic region (23.1%) of the US, followed by the Middle Atlantic (16.4%), and East North Central (14.9%); and was lowest in the Mountain (3.7%), and New England (2.2%) regions as represented in Table 2.

**Table 2:** Geographic variation depicting prevalence of cerebrovascular disease-related hospitalization in vasculitis

Geographical region	Prevalence (%)
New England	2.2
Middle Atlantic	16.4
East North Central	14.9
West North Central	6.0
South Atlantic	23.1
East South Central	10.4
West South Central	10.4
Mountain	3.7
Pacific	12.7

Through this study, major loss of function has been recognized to be more predominant in vasculitis inpatients with CVD (79.9% vs. 69.7%). The mean LOS was 12.9 days vs. 8.1 days and the mean total charges were $169,440 vs. $111,537 in CVD compared to the non-CVD cohorts. In terms of disposition of the vasculitis patients, a higher proportion of patients in the CVD cohort were transferred to a short-term hospital (8.2% vs. 4.6%), and SNF/ICF (22.4% vs. 12%). Also, 3.7% of in-hospital deaths were seen in CVD inpatients with vasculitis which was higher compared to non-CVD (3.5%) as shown in Table 3.

**Table 3:** Differences in hospitalization outcomes in vasculitis inpatients

Variable	CVD (no) in %	CVD (yes) in %	Total	P-value
Severity of illness, in %				
Minor loss of function	4.6	2.2	4.5	<0.001
Moderate loss of function	25.7	17.9	25.5	
Major loss of function	69.7	79.9	69.9	
Other outcomes				
Mean LOS in days	8.1	12.9	-	<0.001
Mean total charges in $	111536.9	169440.4	-	<0.001
Disposition in %				
Routine	63.3	50.7	62.9	<0.001
Transfer to short-term hospital	4.6	8.2	4.7	
Transfer to facility	12.0	22.4	12.3	
Home health care	13.8	12.7	13.8	
Against medical advice	2.8	2.2	2.	
Died in hospital	3.5	3.7	3.5	

Age was not a statistically significant predictor of the risk for CVD in vasculitis inpatients. Females with vasculitis had an increased likelihood of CVD (OR 1.32, 95% confidence interval (CI) 1.12-1.56) compared to the males. Also, Black people had a higher likelihood of CVD (OR 1.32, 95% CI 1.09-1.61) compared to whites for CVD. The comorbidities associated with an increased likelihood of CVD in hospitalized patients were lymphoma (OR 2.46, 95% CI 1.44-4.19), complicated hypertension (OR 1.31, 95% CI 1.11-1.55), and PVD (OR 1.54, 95% CI 1.27-1.87). The comorbidities associated with a lower likelihood were arthropathies (OR 0.60, 95% CI 0.49-0.74), diabetes with complications (OR 0.65, 95% CI=0.52-0.82), and obesity (OR 0.79, 95% CI 0.64-0.97) as shown in Table 4.

**Table 4:** Risk factors for cerebrovascular disease-related hospitalization in vasculitis

Variable	Odds ratio	95% Confidence interval		P-value
		Lower limit	Upper limit	
Age at admission				
18-35 years	1.0			
36-50 years	1.25	0.99	1.56	0.053
51-65 years	0.84	0.68	1.05	0.118
Sex				
Male	1.0			
Female	1.32	1.12	1.56	<0.001
Race/ethnicity				
White	1.0			
Black	1.32	1.09	1.61	0.005
Hispanic	1.09	0.87	1.36	0.477
Other (Asians, Native Americans)	0.64	0.44	0.93	0.020
Comorbidities				
None	1.0			
Arthropathies	0.60	0.49	0.74	<0.001
Lymphoma	2.46	1.44	4.19	<0.001
Metastatic cancer	0.87	0.46	1.64	0.660
Diabetes with complications	0.65	0.52	0.82	<0.001
Hypertension, complicated	1.31	1.11	1.55	<0.001
Obesity	0.79	0.64	0.97	0.023
Drug abuse	0.76	0.57	1.01	0.062
Peripheral vascular diseases	1.54	1.27	1.87	<0.001
Severity of illness, in loss of function				
Minor	1.0			
Moderate	2.04	1.18	3.52	0.010
Major	3.44	2.03	5.83	<0.001

## Discussion

There are very few studies so far that have looked at the prevalence of CVD in vasculitis patients. The prevalence of CVD in GCA was reported to be around 3% to 7% whereas in TA was 13% [19,20]. In our study, we found the overall prevalence of CVD at 2.57% in vasculitis inpatients. A study consisting of patients exclusively with TA found they had 4.6 times the risk of experiencing stroke [21].

Despite a predominantly elderly population in our sample when compared with the non-CVD cohort, the CVD cohort constituted more middle-aged adults with vasculitis. Vasculitis is known to occur at the end of the age spectrum [22]. However, like other studies, we found that age is not a significant predictor of the risk of CVD-related hospitalization in vasculitis patients. We found females at greater risk of developing CVD with vasculitis. There has been limited data on age and sex, and their impact on CVD with vasculitis. However, a review conducted by Katherine et al. found a higher proportion of vasculitis in females which explains the higher risk of CVD in women with vasculitis [23].

In general, vasculitis has multiorgan involvement depending on the type and its presentation varies in different age groups [24]. Polyarteritis nodosa, which is primary medium-sized vessel vasculitis, damages the peripheral nervous system more than CNS and often has complications such as intracranial aneurysm leading to cerebrovascular accidents [24]. It can present as hemorrhagic as well as ischemic stroke as a result of vasculitis itself that may be additionally superimposed by other comorbidities such as hypertension [24]. However, GCA more frequently presents as vision loss than transient ischemic attack and involves mainly vertebrobasilar arteries; strokes with GCA are rare [24]. Takayasu arteritis is much more common in females and Asians; while CVD is rare, when present it is secondary to renal hypertension [24]. The incidence rate of cerebral vasculitis is extremely low in rheumatoid arthritis patients, ranging from 1% to 8% and there is a rare occurrence of CNS vasculitis in systemic lupus erythematosus, and dermatomyositis, sarcoidosis, Behcet’s disease, and eosinophilic granulomatosis with polyangiitis [25]. This could explain our finding of a low association of comorbid arthropathies among CVD patients with vasculitis.

An interesting finding of our study was a greater association of lymphoma in patients with vasculitis and CVD. A study done at the Mayo clinic identified an association between lymphoma in primary CNS vasculitis patients [26]. This finding is suggestive of involvement underlying immunologic paraneoplastic etiology [26]. They also identified a higher disability at the last follow-up among these patients with PACNS and lymphoma, especially those diagnosed in the latter part of their life [26]. Among other comorbidities in our cohort, patients with hypertension and vasculitis were more likely to suffer from CVD. A retrospective analysis established that vasculitis was the most common cause of secondary malignant hypertension, and the diagnosis was often missed by clinicians [27]. 

In our study the hospitalization cost of patients with vasculitis and CVD was 34% higher than those without CVD. Advanced vasculitis can be aggressive and additional CVD can impose a greater burden on the cost of care [21]. In terms of geographical prevalence, CVD in vasculitis patients was more prevalent in the South Atlantic region which is part of the famous “stroke belt” in the US [28]. This geographical region is also known for the highest stroke mortality [28]. It was concluded in 2017 that 26% of residents of the “stroke belt” were comprised of the non-Hispanic Black population [28]. Along similar lines, our study has found a higher likelihood of CVD in Black people compared to white patients with vasculitis. Another potential contributor to the higher prevalence in the South Atlantic region was a higher prevalence of comorbidities such as hypertension and diabetes [28].

A major strength of our study can be derived from its large population-based data set and is the NIS representative of all patients with vasculitis in the US. To the best of our knowledge, this is the first study looking at epidemiological risk factors of CVD among all vasculitis inpatients. Clinical presentation of CNS vasculitis can be variable and difficult to diagnose. Hence there may be underreporting of the disease. And given the nature of the NIS data set, we could not delineate the differences in prevalence in vasculitis subtypes.

## Conclusions

The prevalence of CVD-related hospitalization in vasculitis patients was 2.5%. Comorbidities including lymphoma, PVD, and hypertension further increase the risk for CVD in patients with vasculitis. Through this study, we found that these at-risk patients have a higher loss of functioning that affects patients’ quality of life (QoL) requiring longer hospitalization stay and thereby increasing healthcare costs and a higher likelihood of increased care post-discharge. Future advances must focus on preventive measures and early diagnosis and treatment of comorbid risk factors that may increase the risk of CVD in patients with vasculitis thereby improving their QoL.
